# Global deregulation of ginseng products may be a safety hazard to warfarin takers: solid evidence of ginseng-warfarin interaction

**DOI:** 10.1038/s41598-017-05825-9

**Published:** 2017-07-19

**Authors:** Haiyan Dong, Ji Ma, Tao Li, Yingying Xiao, Ning Zheng, Jian Liu, Yu Gao, Jingwei Shao, Lee Jia

**Affiliations:** 1 Cancer Metastasis Alert and Prevention Center, and Pharmaceutical Photocatalysis of State Key Laboratory of Photocatalysis on Energy and Environment, College of Chemistry, Fuzhou University, Fuzhou, 350116 China; 2 Fujian Provincial Key Laboratory of Cancer Metastasis Chemoprevention and Chemotherapy, Fuzhou University, Fuzhou, 350116 China

## Abstract

Recent global deregulation of ginseng as the table food raises our concern about the possible ginseng-warfarin interaction that could be life-threatening to patients who take warfarin for preventing fatal strokes and thromboembolism while using ginseng products for bioenergy recovery. Here we show that quality-control ginsenosides, extracted from ginseng and containing its major active ingredients, produce dose- and time-dependent antagonism in rats against warfarin’s anti-coagulation assessed by INR and rat thrombosis model. The interactions between ginsenosides and warfarin on thrombosis, pharmacokinetics, activities of coagulation factors and liver cytochrome P450 isomers are determined by using thrombosis analyzer, UPLC/MS/MS, ELISA and real-time PCR, respectively. The antagonism correlates well with the related pharmacokinetic interaction showing that the blood plateaus of warfarin reached by one-week warfarin administration are significantly reduced after three-week co-administration of warfarin with ginsenosides while 7-hydroxywarfarin is increased. The one-week warfarin and three-week warfarin-ginsenosides regimen result in restoring the suppressed levels by warfarin of the coagulating factors II, VII and protein Z, and significantly enhance activities of P450 3A4 and 2C9 that metabolize warfarin. The present study, for the first time, provides the solid evidence to demonstrate the warfarin-ginsenoside interaction, and warns the warfarin users and regulation authorities of the dangerous interaction.

## Introduction

The market of dietary supplements (or nutraceuticals) and botanical drugs reached great monetary success, and the sale in the US was about $86 billion, and in Asia, $210 billion in 2008^1^. Among the top sellers, ginseng is the most-consumed dietary supplement worldwide. It is taken as a nutraceutical adaptogen or nourishing stimulant for thousands of years^2^. To reevaluate the millennium-old phytomedicine from the modern biomedicine, we recently comprehensively overviewed etymology and pharmacognosy of ginseng, including its natural origin, physical appearance and specie classification. Government regulations cannot completely scrutinize all the global ginseng markets and ginseng products. Although cultivation and processing procedures may change metabolism profiles of ginseng’s active ingredients and the related effectiveness, standardized analytical methods have been developed for quality control of various ginseng products^3^. We also summarized about 107 chemical entities separated from the roots, leaves and flower buds of ginseng family and categorized these entities into about 18 groups based on their structural similarity^4^. Modern biomedicine verifies that ginseng’s antihyperglycemic effect benefits type II diabetics^5^; its well-known aphrodisiac effect enhances sex performance of both genders; its cardiovascular effect strengthens cardiac contraction and may cause hypertension in some sensitive populations^6^. Its cognitive and neuropharmacological effect tested in various rat models may help cognition its uses as an adjuvant, anti-inflammatory, or immunotherapeutic agent may boost immune activity, appetite and life quality of patients during their chemotherapy, radiation, and recovery after serious surgery^7^.

Warfarin is the most clinically-prescribed anticoagulant for treatment or prevent of blood clots in veins (deep vein thrombosis) and arteries, pulmonary embolism, chronic atrial fibrillation, and myocardial infarction^8, 9^. However, its therapeutic window determined by the international normalized ratio (INR) is narrow to 2.0–3.0, and its interactions with many drugs, dietary supplement, and even foods could be deadly if the INR level raises over the therapeutic window because any blood clot could occur due to the interaction, which could cause a life-threatening consequence, specifically to those mechanical bileaflet aortic valve prosthesis patients and many others who have just completed cardiovascular operation and are under important warfarin anticoagulation^10, 11^. During the period, any drugs, dietary supplement, or even foods that upregulate activity of the hepatic P450 enzyme may shorten the half-life of warfarin and result in deadly blood clots^10^.

Patients often feel tired and weakened, and would like to take the well-known adaptogen or stimulant ginseng for boosting their body energy for quick recovery. A few case reports revealed the dangerous decrease in patient’s INR who had been under warfarin anticoagulation for a long period but took ginseng products recently before the INR decrease. These cases resulted in hospital emergency^12, 13^. Although laboratory studies investigated the possibility of ginseng-warfarin interaction by using rats^14^, healthy volunteers^15^, ischemic stroke patients^16^, or patients with cardiac valve replacement^17^, those studies are somewhat misleading and confusing for the following reasons. Therefore, these investigation results cannot be conclusive: 1. Lacking quality control of the tested ginseng products; 2. Not giving the blood warfarin enough time (usually 3–5 days) to maximize its effect by degradating the existing vitamin K-dependent clotting factors before giving the tested ginseng products; and 3. Not connecting blood concentrations of warfarin with its altered efficacy.

Recently, ginseng products are deregulated to table foods or nutraceuticals in Asia countries such as China, Korea, and Japan. The action is boosting the ginseng industry and its related up and down economic chains in these countries^18^. Although the deregulation and less red tape are the good news for the ginseng industry to celebrate, the news raised our concern about the possible life-threatening ginseng-warfarin interaction^10, 19^, we therefore started the well-designed study to investigate possibility of the interaction. The study reported below presented the solid evidence to confirm the interaction for the first time, and caution the warfarin-takers of potential ginseng interruption.

## Results

### Quality control analysis of ginsenosides

**Figure 1:**
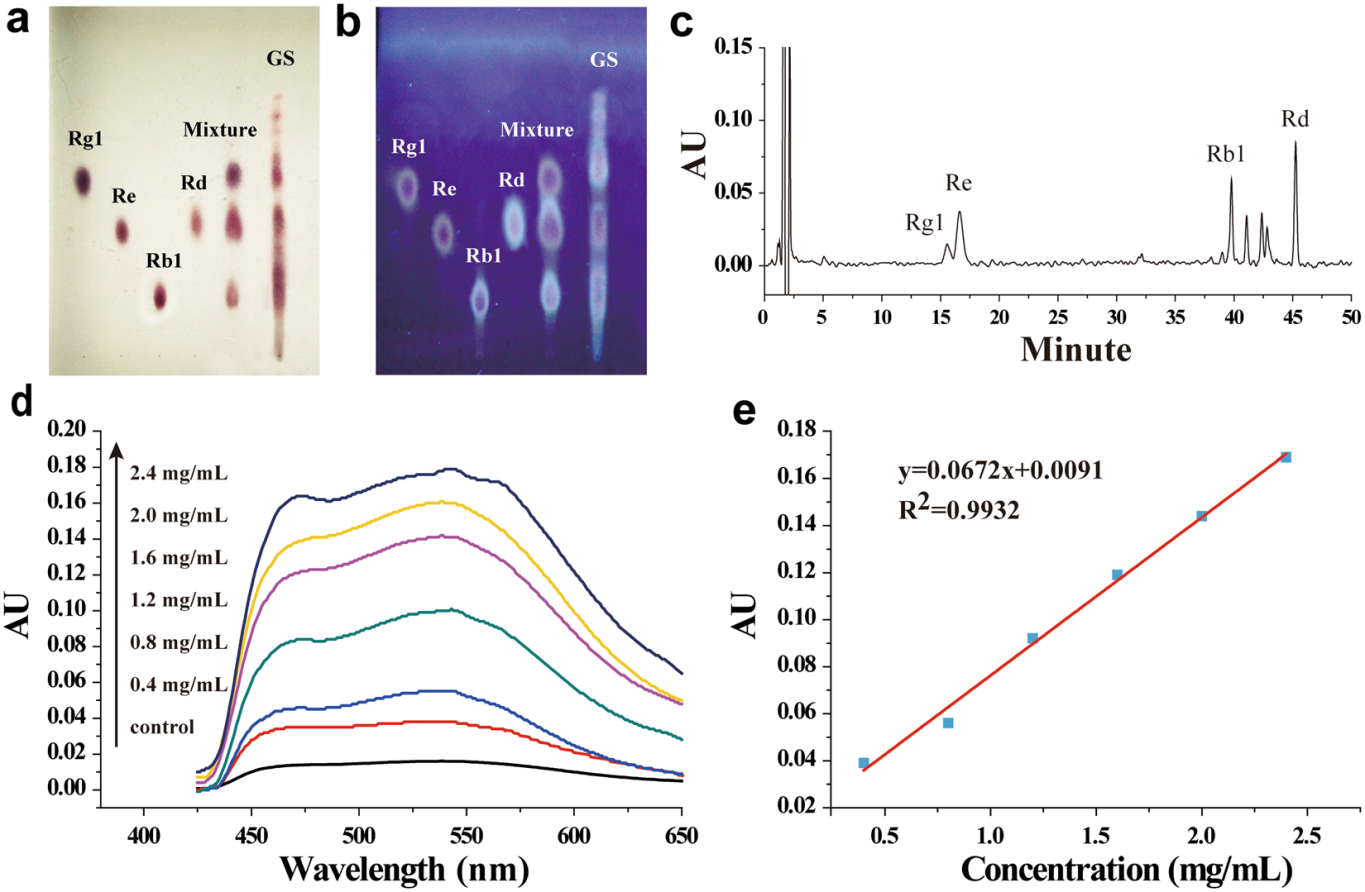
Quality control analysis of the most-consumed ginsenoside powder. (**a**,**b**) The quality control solutions of purified ginsenosides (GS), and standardized ginsenoside component Rg1, Re, Rb1, and Rd were pipetted on to SiO_2_-coated thin layer chromatography plates, respectively, and developed with the mobile phase followed by chromogenic stain (10% H_2_SO_4_), and sunlight (**a**) and UV exposures (**b**). The results demonstrated that the test GS contained Rg1, Re, Rb1, and Rd. (**c**) HPLC chromatograms showed the peaks and retention times of each active component from the purified GS. (**d**) Scanning of ultraviolet spectra of various concentrations of ginsenoside Re that is used as the quality control standard as regulated by the latest Chinese Pharmacopeia. (**e**) The standard curve of ginsenoside Re generated to calculate the relative purity of GS.

Previous publications on ginseng pharmacological effects often lack necessary quality control analysis of the tested ginseng products, which makes it difficult to reproduce the reported pharmacological effects of the products and verify these effects in human^3, 4^. To provide the robust reproducible data to demonstrate the interaction between ginseng and warfarin, we prepared the quality control solutions of ginsenosides (GS) and Rg1, Re, Rb1 and Rd, respectively, and pipetted drops of the GS sample in parallel with single Rg1, Re, Rb1 or Rd component on thin layer chromatography plates, and developed these tested materials on the plates to examine their quality. Fig. 1a,b showed that the GS sample (right lane) contained components that had the same retention time as Rg1, Re, Rb1 and Rd showed on the plates under sunlight and UV exposures, indicating that GS contained at least four active ginseng components^20^. The result was later reproduced by using the analytical HPLC method (Fig. 1c), which showed that the four components appeared at their corresponding retention time points determined by using pure Rg1, Re, Rb1 or Rd, separately.

Following the guidance of the provision of the Pharmacopoeia of the People’s Republic of China (the 2010 edition), which requires the use of ginsenoside Re as the quality control standard for ginseng products, we developed the quantitative HPLC method to analyze the concentrations of Re (Fig. 1d). The UV-vis absorbance versus each Re concentrations was linear from Re 0.4 to 2.4 mg/mL with the correlation coefficient (R^2^) 0.9932 for the standard curve (Fig. 1e). According to the fitting curve, we back-calculated that our GS contained Re 81.95 (mean %), which fulfilled well the 65–85% of Re required by the China Pharmacopoeia for ginseng products in terms of their quality control. Therefore, the results obtained from the batch of GS were reliable.

### Time- and dose-dependent changes in rat INR caused by warfarin and GS

**Figure 2:**
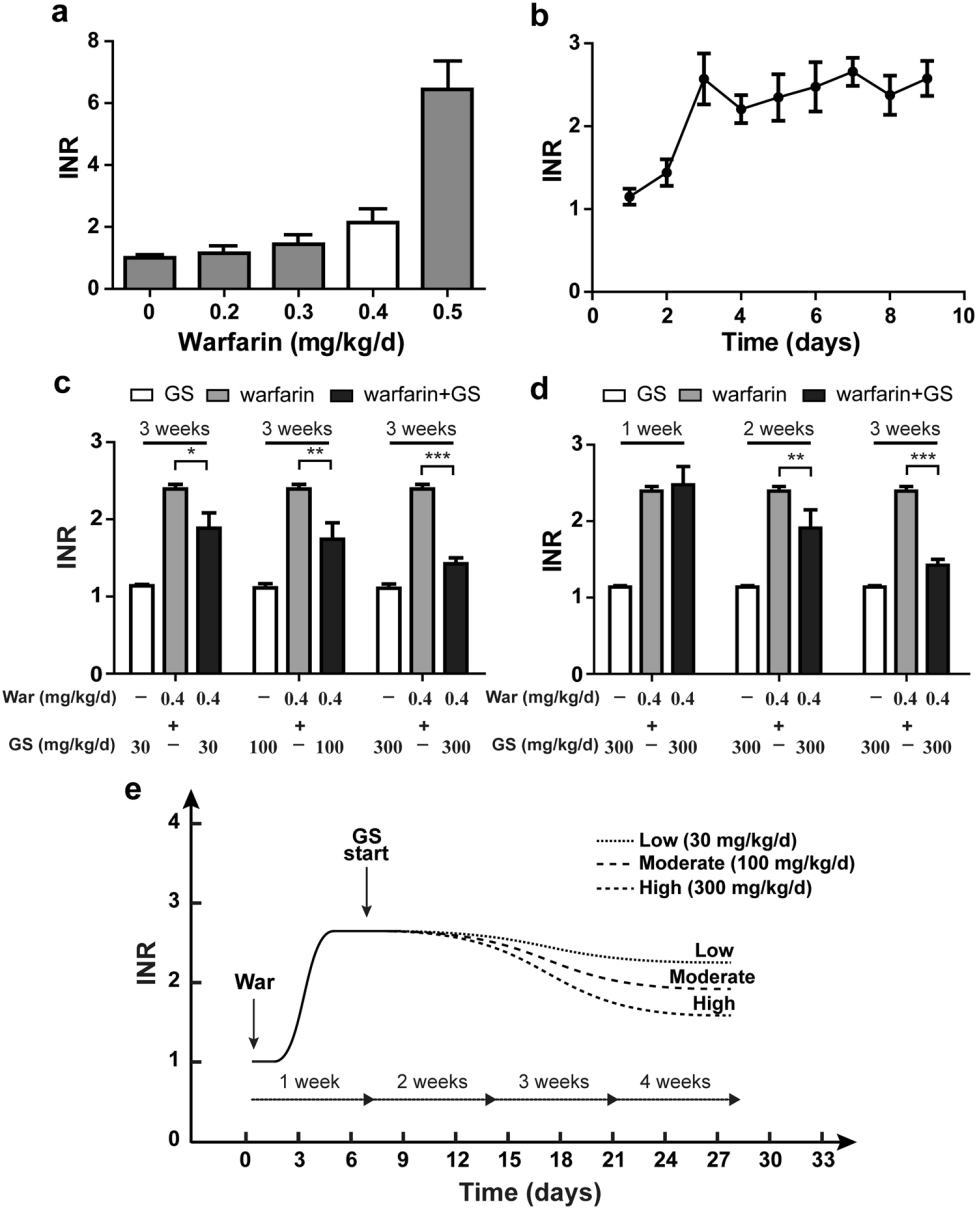
Dose-dependent and time-dependent anti-coagulation of warfarin administered alone or with GS to rats. (**a**) Dose-dependent anti-coagulation of warfarin on rat INR that was measured following 7 consecutive administration of oral warfarin, and the dose 0.4 mg/kg/day was determined as the optimal dose, and used to monitor the dynamic change in INR in rats (**b**). (**c**) Changes in INR by three weeks of warfarin (0.4 mg/kg/day), or GS (30, 100 and 300 mg/kg/day), or warfarin combined with GS. Note, GS antagonized the effect of warfarin dose-dependently. (**d**) Time-dependent antagonism by GS against warfarin anti-coagulation was more significant by 3-weeks GS than by 1-week GS. (**e**) Dynamic change in rat INR by warfarin followed by three doses of GS. Data represent mean ± SD (n = 10 rats per group), *P < 0.05, **P < 0.01, ***P < 0.001.

To verify whether there is interaction between warfarin and GS in coagulation, we first explored the International Normalized Ratio (INR) and dose relationship of warfarin in order to determine its optimal dose that could produce anti-coagulation effect with a good safety profile. Based on the equivalent surface area dosage conversion factors from human to rat^21^, we first designed four doses of warfarin for its oral administration to rats with the hope that we could find one dose that could be both anti-coagulant and safe to rats. Fig. 2a showed that the dose 0.4 mg/kg/day (7 days) seemed to be optimal because this dose significantly increased rat INR without causing obvious hemorrhage as observed in group of 0.5 mg/kg/day. The dynamic change in rat INR following oral warfarin at 0.4 mg/kg/day (Fig. 2b) exhibited that the INR was gradually increased and reached its peak on day 3 or 4, and kept the plateau level as long as warfarin was dosed. Hence, the dose of 0.4 mg/kg warfarin was used throughout the study unless otherwise stated.

We then investigated whether GS could interrupt warfarin-caused increase of INR. As shown in Fig. 2c, three-week administration of warfarin alone to rats at 0.4 mg/kg/day significantly enhanced the INR level. Co-administration of GS (30, 100 and 300 mg/kg/day) with warfarin for 3 weeks following 1-week administration of warfarin to the rats produced dose-dependent antagonism by GS against warfarin-enhanced INR. The GS doses chosen were based on the most-consumed human GS doses^4, 7^. We then used the GS most-effective dose 300 mg/kg/day to match the time-course of GS intervention on the INR enhanced by warfarin. Fig. 2d showed that the intervention took time to become significant, arguing against previous reports that there were no ginseng-warfarin interaction^14, 16^. Fig. 2e described the time windows and related dynamics of warfarin and GS needed to make a change in the INR index.

### Warfarin-GS interaction in K-carrageenan-induced rat thrombosis model

**Figure 3:**
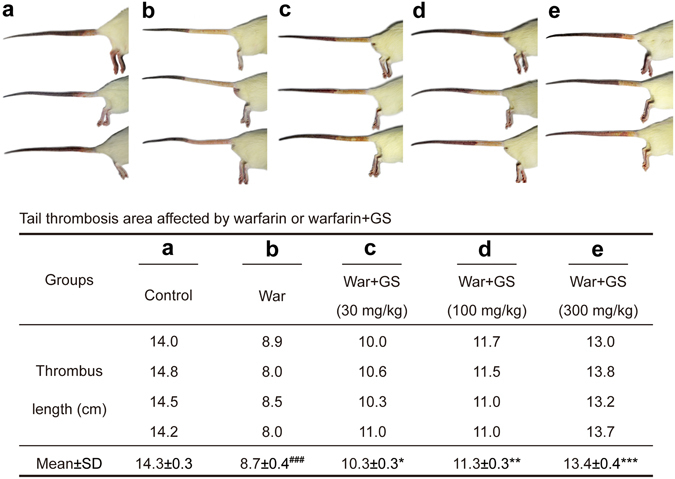
Effects warfarin alone, or administered with GS on rat tail thrombosis induced by K-carrageenan. Upper panel, the tail thrombosis induced by K-carrageenan (**a**) and attenuated by one-week warfarin (0.4 mg/kg/day) (**b**); (**c**–**e**) one-week warfarin and three-week warfarin co-administered with GS (30, 100 and 300 mg/kg/day) produced GS dose-dependent antagonism on warfarin’s anti-coagulation effect. Lower panel shows the related quantitative analysis. ^###^ *P* < 0.001, statistically significant difference compared with group A; *P < 0.05; **P < 0.01; ***P < 0.001 compared with group B (n = 4 per group).

The above study demonstrated pharmacological interaction between warfarin and GS using the INR as the thrombosis index. Here we used the well-established K-carrageenan-induced rat tail thrombosis model^22, 23^ to further investigate whether GS intervenes the warfarin’s anti-coagulation effect. As shown in Fig. 3, pretreatment of the rats with warfarin for one week significantly reduced the thrombosis area caused by tail injection of K-carrageenan in comparison with the control group. One-week warfarin followed by 3-week co-administration of warfarin with GS of 3 doses resulted in attenuation by GS of the warfarin’s anticoagulation as evidenced by the significant extension of the already-shrunk infarcted area after warfarin treatment. The antagonism by GS against warfarin’s anti-coagulation appeared to be GS dose-dependent (Fig. 3).

### Pharmacokinetic intervention by 3-week GS on a single dose of warfarin

**Figure 4:**
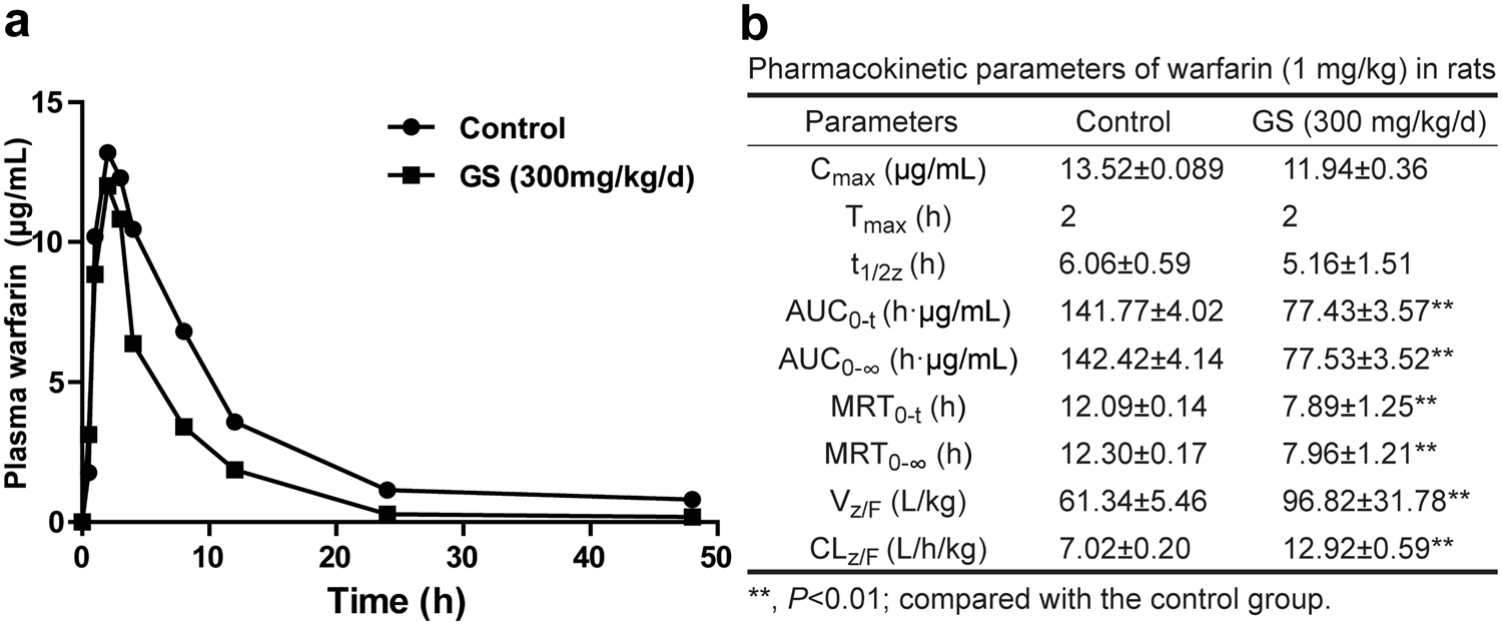
Plasma warfarin concentration-time curve (left) and the related pharmacokinetic parameters (right). A single dose of warfarin (1 mg/kg) was given to rats pretreated with GS (300 mg/kg/day, n = 10) for 3 weeks.

Although the above data demonstrated the pharmacodynamical intervention by GS on warfarin-induced changes in INR and infarcted area, we still wondered if GS could cause a change in pharmacokinetic profile of warfarin. We therefore administered GS to rats at 300 mg/kg/day for 3 weeks, and then gave a single dose of warfarin (1 mg/kg) to both the GS-pretreated rats and the untreated control rats, respectively. We withdrew the rat blood at the designated time points to measure the blood concentrations of warfarin by using the UPLC/MS/MS method considered good practice^21, 24^. Fig. 4a showed that plasma concentration level of warfarin, i.e., the warfarin plasma area under curve (AUC) after a single oral administration of warfarin, was reduced if the rats were already pretreated with GS at 300 mg/kg/day for 3 weeks. Fig. 4b showed the statistically significant decrease in warfarin AUC and other pharmacokinetic parameters after the 3-week GS pretreatment. The results clearly indicated that pretreatment of rats with GS for 3 weeks reduced plasma warfarin level of a single administration of warfarin.

### Changes in plasma warfarin and 7-OH-warfarin after repeated warfarin administrations with or without GS

We next determined if GS intervened plasma warfarin after repeated administrations of warfarin. We first administered warfarin to rats for one week, allowing warfarin to reach its plasma plateau (Fig. 2b). The rats were then divided into four groups, one receiving warfarin alone, others receiving warfarin plus 3 doses of GS, separately. The blood was withdrawn at 3 hr after warfarin administration in order to measure plasma warfarin and 7-OH-warfarin levels^25^.

**Figure 5:**
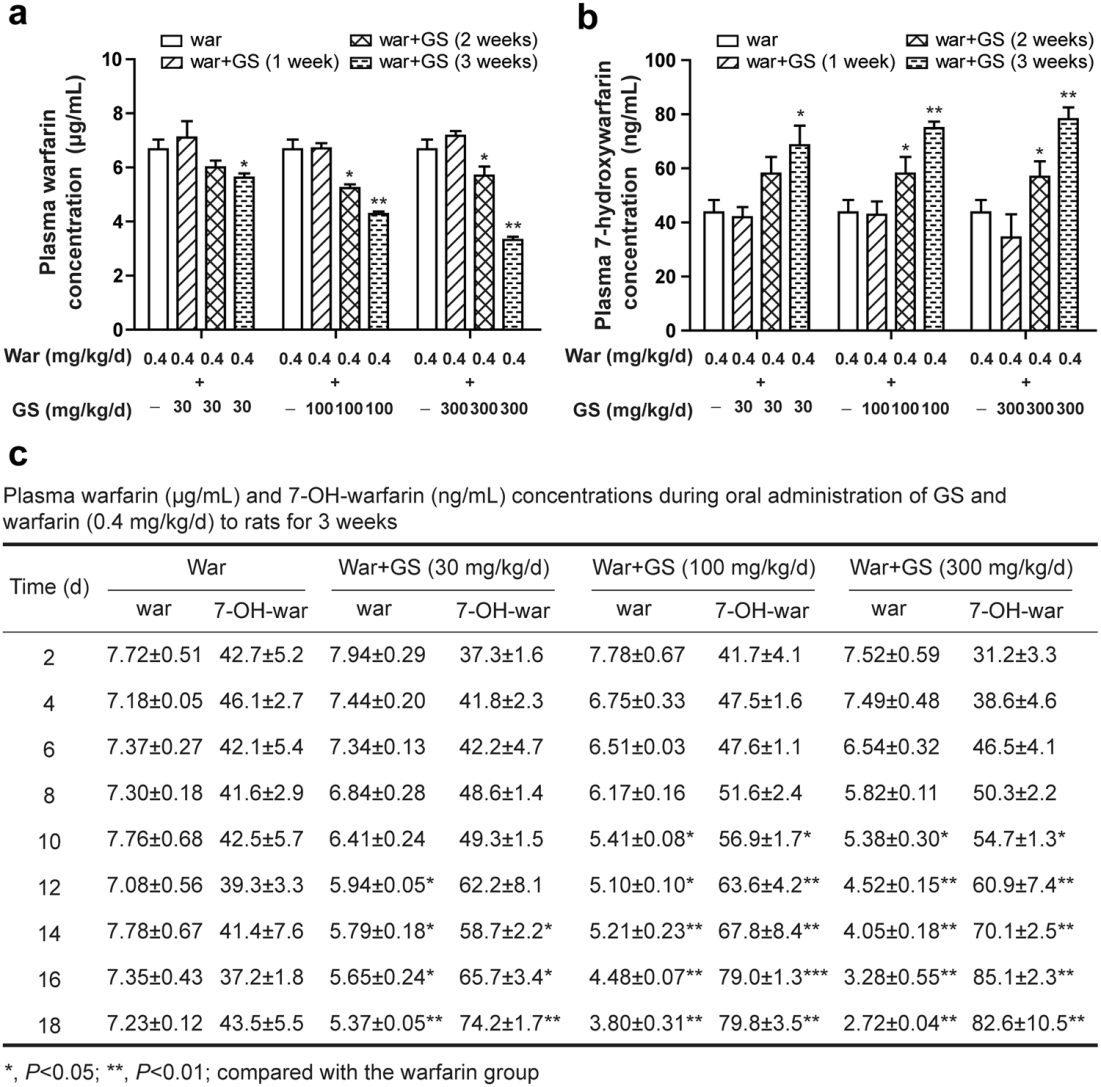
GS dose- and time-dependent intervention on plasma warfarin concentrations. (**a**) One-week warfarin (0.4 mg/kg/d) plus three-week warfarin co-administered with GS (30, 100, 300 mg/kg/day) for 1, 2, or 3 weeks decreased plasma warfarin (in µg/mL) in a dose- and time-dependent manner; (**b**) meanwhile, the regimen increased plasmawarfarin metabolite 7-OH warfarin (in ng/mL); (**c**) quantitative data showed the concomitant decrease in plasma warfarin and increase in 7-OH warfarin. The blood was withdrawn from rat tail vein for UPLC-MS/MS analysis every other day. *P < 0.05; **P < 0.01, statistically significant differences between warfarin alone and warfarin plus GS (n = 10 per group), unless otherwise stated.

One week of co-administration of warfarin with GS did not significantly change the plasma concentrations of warfarin (Fig. 5a). However, after two consecutive weeks of the co-administration, plasma warfarin level was significantly reduced when GS was given at 100 and 300 mg/kg/day. More significant reduction in plasma warfarin was found when the co-administration prolonged to 3 weeks, and the reduction in plasma warfarin was GS dose-dependent.

Since warfarin is primarily metabolized to 7-OH-warfarin, we used the well-established UPLC/MS/MS method to simultaneously measure 7-OH-warfarin (see supplementary information). Interestingly enough, with the decrease of plasma warfarin, the plasma 7-OH-warfarin level was increased, instead (Fig. 5b), and the increase of 7-OH-warfarin was dependent on GS treatment time and dose. Fig. 5c quantitatively showed the decreased plasma level of warfarin and the increased plasma 7-OH-warfarin in parallel. The result suggests the *in vivo* conversion of warfarin to 7-OH-warfarin induced by GS.

### Antagonism by GS against warfarin anti-coagulation at molecular level

**Figure 6:**
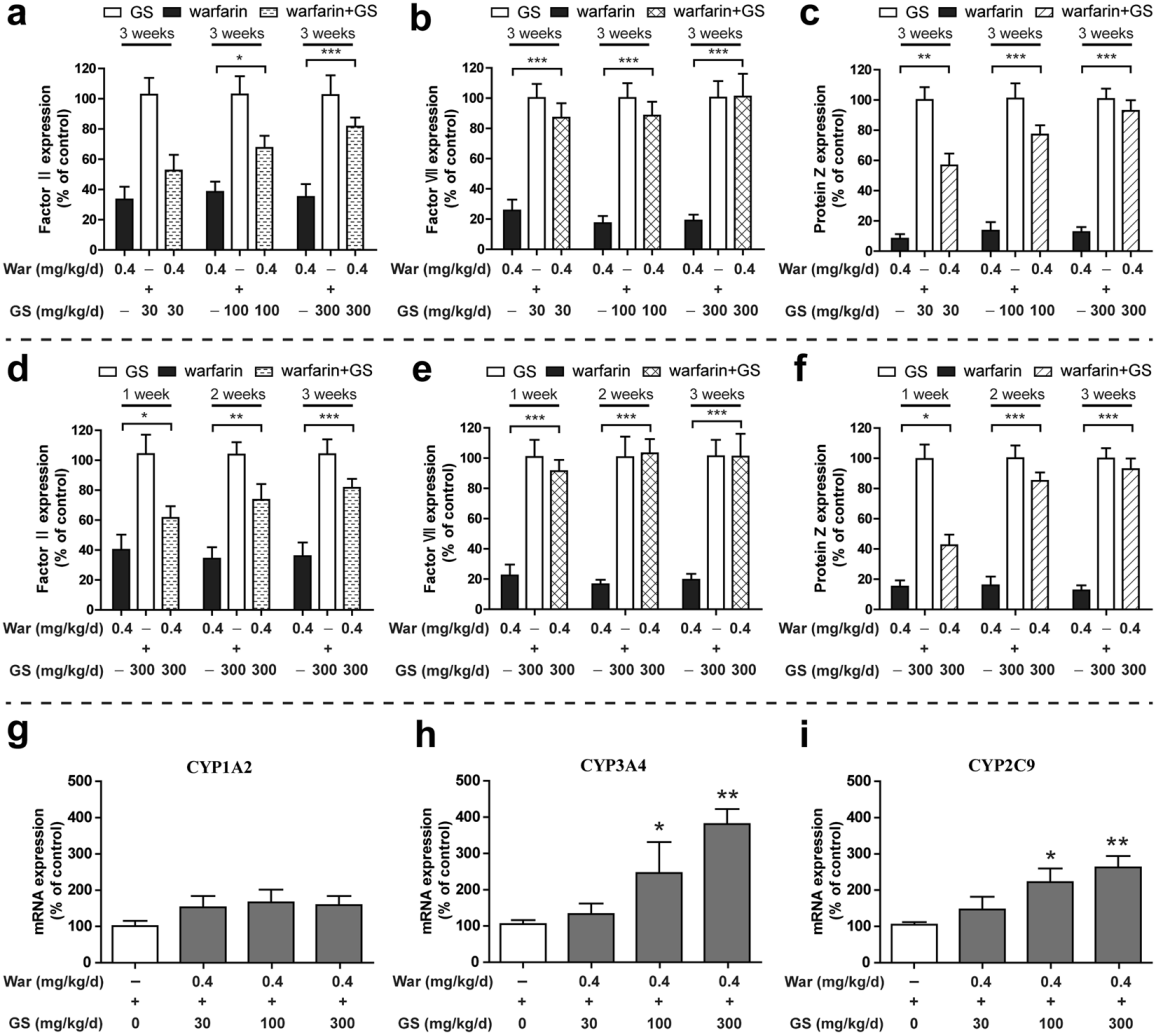
GS antagonized inhibition by warfarin of coagulation factors II, VII and protein Z, and enhanced activity of cytochrome P450 3A4 and 2C9 following one-week warfarin plus three-week warfarin co-administered with GS. (**a**–**c**) GS dose-dependent (30, 100 and 300 mg/kg/day for 3 weeks; (**a**–**c**), and time-dependent (300 mg/kg/day for 1, 2 and 3 weeks; (**d**,**e**) antagonism on inhibition by warfarin of coagulation factors II, VII and protein Z; (**g**–**i**) dose-dependent upregulation by GS (30, 100 and 300 mg/kg/day) on mRNA expression of rat hepatic cytochrome P450 CYP1A2, 3A4 and 2C9. All rats were pretreated with oral warfarin for 1 week followed by the co-administration regimen. Data represent the mean ± SD (n = 10 per group); *P < 0.05; **P < 0.01; ***P < 0.001, compared with warfarin alone (**a**–**f**), or the untreated controls (**g**–**i**).

**Figure 7:**
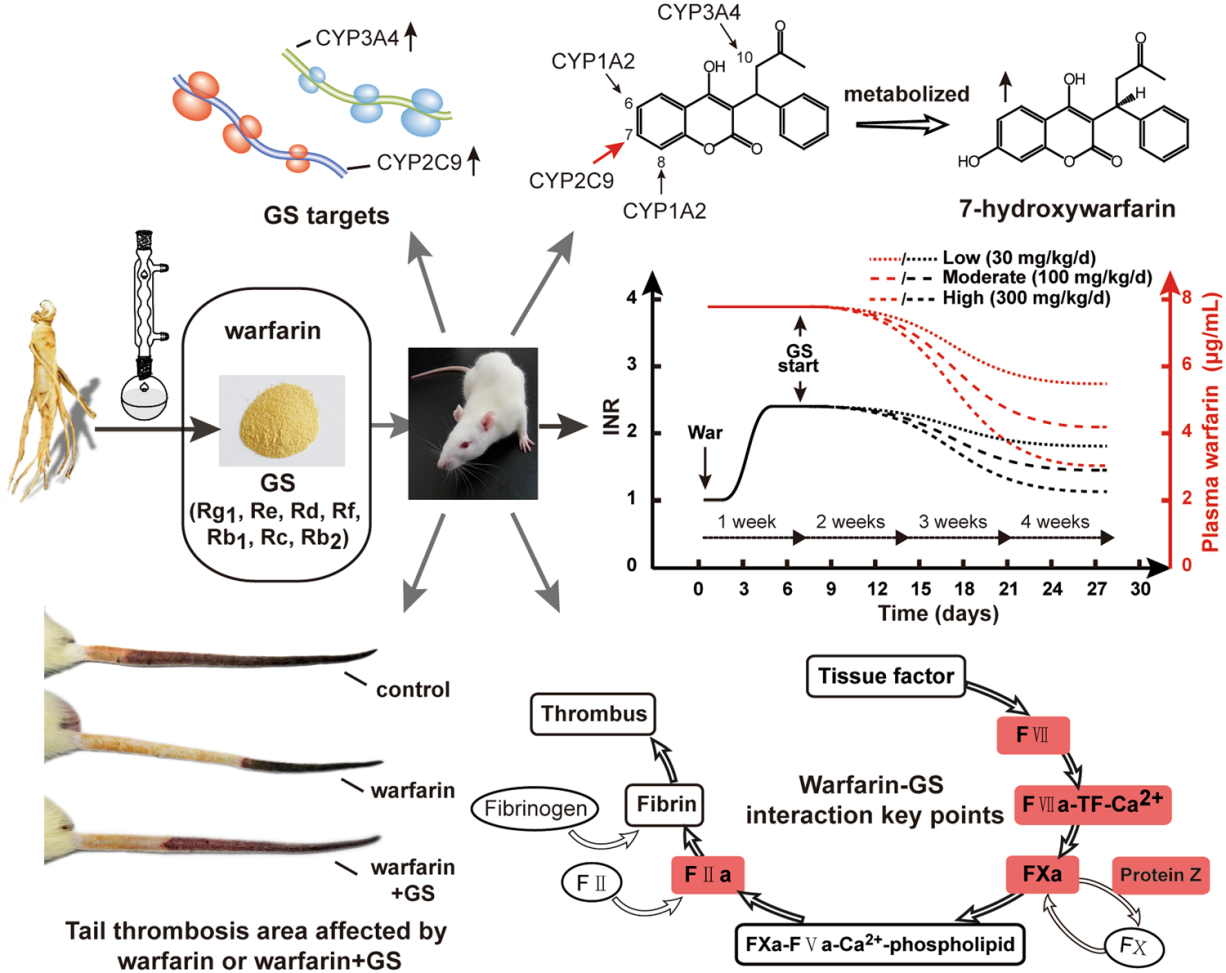
Schematic of the investigation on warfarin-GS interaction and related molecular mechanisms. GS extracted from ginseng contained active components, and was administered to rats based on 1-week warfarin followed by 3-weeks of warfarin plus GS (30, 100, and 300 mg/kg/day) regimen, which reversed the enhanced INR and attenuated rat tail thrombosis caused by warfarin-pretreatment. GS intervention on warfarin anticoagulation is clearly GS dose- and time-dependent, which involves restoring warfarin-downregulated coagulation factor VII, II, and protein Z, and activating liver cytochrome P450 CYP2C9 and 3A4. The former event facilitates coagulation, and the latter metabolizes warfarin leading to attenuated anticoagulation.

The above pharmacodynamics and pharmacokinetics correlation clearly demonstrated the interruption by GS on warfarin anti-coagulation effect. Next we wanted to find out the molecular targets that both GS and warfarin antagonize et. We pretreated the rats with warfarin for 1 week, and then co-administration of warfarin with GS at 30, 100 and 300 mg/kg/day to the rats for 3 weeks. The blood samples were withdrawn and the coagulation factors II, VII and protein Z were measured by the ELISA method^26, 27^. The administration regimen revealed that warfarin treatment suppressed the activity of the three coagulation factors. Addition of GS to warfarin treatment restored the warfarin-suppressed activity of the three coagulation factors in a GS dose-dependent manner (Fig. 6a–c), among them factor II and protein Z showed obvious GS dose-dependent effect, and factor VII reacted fast to GS stimulation, and the reaction completed within a day after addition of GS to the regimen, probably because the factor VII is positioned at the starting point of the coagulation reaction chain (Fig. 7). As a result, a quick response (instead of a linear one) was observed with factor VII (Fig. 6b,e). Fig. 6d–f showed time-dependent antagonism of GS (300 mg/kg/day) on warfarin-suppressed activity of factors II, VII and protein Z. The results indicated that it took time for those suppressed factors to be restored. Therefore, the conclusions from previous studies that did not give enough time for the interaction reaction to be completed could not be accurate, and should be re-evaluated^14, 16^.

Liver microsomes contain various cytochrome P450 enzymes (CYP450) that are responsible for drug metabolism^28^. In order to identify the CYP450s that are involved in warfarin-GS interaction, we treated rats with warfarin plus 3 doses of GS (30, 100, 300 mg/kg/day) for 3 weeks, and then took liver tissue for preparation for hepatic cells. We extracted the total RNA from the cells, and examined mRNA expression of the related CYP450s. Fig. 6g–i clearly showed the upregulation by GS of CYP2C9 and 3A4. CYP2C9 is responsible for metabolism of warfarin to 7-OH-warfarin that was well detected in the present study (Fig. 5), and CYP3A4 is responsible for metabolism of warfarin to 10-OH warfarin^25^.

## Discussion

In the present study, we use quality-controlled GS powder extracted from ginseng, which contained major ginseng active ingredients, to demonstrate that GS produced dose- and time-dependent antagonism in rats against warfarin’s anti-coagulation evidenced by the changes in INR (Fig. 2) and rat tail thrombosis area (Fig. 3) in the presence and absence of GS. The pharmacological antagonism between ginsenosides and warfarin takes at least one week to become obvious, and the results obtained from three-week co-administration of GS and warfarin at a higher dose are more significant than those from a shorter time period and a lower dose. The K-carrageenan-induced rat tail thrombosis model is the commonly-used thrombosis model that simulates well with the human infarction process^22^.

The pharmacological antagonism-correlates well with the pharmacokinetic interaction between ginsenosides and warfarin. Using the robust and considered good-practice pharmacokinetic methods^21, 28^, we further demonstrated that when GS antagonized warfarin’s anti-coagulative effect, it also reduced blood concentration of warfarin (Fig. 7), and the time course of the changes caused by GS in warfarin’s anti-coagulation synchronized with the changes caused by GS in warfarin’s blood concentrations. The pharmacokinetic interaction between ginsenosides and warfarin is also GS dose- and time-dependent. The disappearance of warfarinin blood coordinated well with the appearance of its metabolite 7-OH-warfarin (Fig. 5). One-week warfarin followed by three-week co-administration of warfarin and ginsenosides of 3 doses restored the suppressed coagulating factors II, VII and protein Z by warfarin. In parallel, the liver drug-metabolism enzyme cytochrome P450 3A4, 2C9, and 1A2 were significantly upregulated by GS in a GS dose-dependent manner (Fig. 6g–i). The changes by GS of the activities of these coagulating factors and cytochrome P450 enzymes elucidate the mechanisms of action by which GS antagonizes the anti-coagulation effect of warfarin (Fig. 7).

In the present study, we used the dose of GS as high as 300 mg/kg/day for 3 weeks and the regimen seems to be fairly tolerable to the rats without significant adverse effects. According to the equivalent surface area dosage conversion factors^21^, the GS dose 300 mg/kg/day can be converted into the human equivalent dose (HED) of 45 mg/kg/day using the formula HED = animal dose in mg/kg × (animal weight in kg/human weight in kg)^0.33^, that is 300 mg/kg × (0.2 kg rat weight/60 kg man weight)^0.33^. For a 60-kg man, the daily GS dose is 2.7 g. People usually consume 10–20 grams of Panax ginseng roots per time, and the total ginsenoside content in Panax ginseng varied from 0.2 to 2% (w/w) for ginseng main roots, equivalent to 0.4 grams of GS^4^. The GS doses used in the present study and the above-calculation indicate the safety dosage range of ginseng and GS.

Warfarin is the vitamin K antagonist, and often used at clinic for preventing strokes, blood clots in veins and arteries, systemic embolic events in patients with atrial fibrillation, and myocardial infarction^29^. Any seemly-insignificant intervention on warfarin’s anticoagulation, no matter if the intervention happens acutely or chronically (the latter is often seen with botanic medicines such as ginseng), could cause a serious consequence to those patients who need stable anticoagulative effect. Often, these patients look for dietary supplements with the hope that these natural “safe” supplements could help their Yin-Yang balance^30^. However, this study presented the solid evidence, obtained from the quality-controlled ginsenosides to molecular coagulation factors and drug metabolism enzymes in conjunction with animal pharmacokinetics and pharmacodynamics, to warn ginseng consumers and regulation authorities of the ginseng-warfarin interaction that may be dangerous to patients who are under daily necessary warfarin anticoagulation.

## Methods

### Materials

Sprague Dawley rats (200 ± 20 g) were purchased from Shanghai Laboratory Animal Center (Shanghai, China). All studies involving animals were carried out in accordance with the NSFC regulation concerning the care and use of experimental animals and approved by the Institutional Animal Care and Use Committee of Fuzhou University to reduce the suffering and use of animals.

### Ginsenosides preparation

GS was prepared as follows: the crude ginseng extract (Nanjing Zelang Biological Technology Co. Ltd, China) was refluxed with 10-time volume of methanol (w/v) at 70 °C for 4 h in water bath. The methanol extract solution was filtered and concentrated by rotary evaporator at 40 °C. The solid extract was resolved in 20-time volume of distilled water, and re-extracted by butanol six times. The GS was concentrated by rotary evaporator at 90 °C. Methanol and acetone were used to make the extract in powder, which was then filtered and made dry under vacuum to obtain the recrystallized GS that was used for preparation of the quality control GS methanol solution^20^.

### Quality control analysis of GS

The quality control analysis of GS was conducted following the guidance of the provision of the Pharmacopoeia of the People’s Republic of China (the 2010 edition). Briefly, the standard methanol solutions of ginsenoside Rg1, Re, Rb1, Rd and GS were pipetted on silca gel (SiO_2_)-coated TLC plates at the same concentration of 1 mg/mL. The plates were developed with the mobile phase (chloroform:ethyl acetate:methanol:water, 15:40:22:5, v/v) for approximately 50 min at room temperature. The plates were sprayed with 10% H_2_SO_4_ as the chromogenic agent, and dried at 105 °C. The results were examined under sunlight and UV lamp at 365 nm.

The Waters 2695 series HPLC equipped with the Waters 2798 UV absorbance detector set at 203 nm and C18 column (5 μm, 4.6 × 5 mm, Sigma Amide) were used for separation of Rg1, Re, Rb1, Rd and GS in methanol. A gradient mobile phase composed of methanol (solvent A) and 0.1% acid water (solvent B) was used according to the following program: isocratic elution with A:B (21:79; v/v) for the first 30 min, and then gradient elution with A:B (40:60; v/v) for the next 20 min at the flow rate of 1.3 mL/min, 30 °C. The injection volume was 20 μL.

The quality control methanol solutions of Re (1.0 mg/mL) and GS (2.0 mg/mL) were prepared as the Re standard solution of 20, 40, 80, 120, 160, and 200 μL, and GS standard solution of 50 μL. The solutions were evaporated to dryness using nitrogen at room temperature. The solutions mixed with 1% vanilline perchloric acid were heated in a water bath at 60 °C for 15 min, then kept in iced water for 2 min, and mixed with 77% sulfuric acid. Absorbance at 540 nm of each sample solution was measured by the UV-Vis spectrophotometry. The standard curve of absorbance versus concentrations of each sample was established to calculate GS concentrations.

### Warfarin anticoagulation experiment

To explore the optimal dosage and anticoagulation efficacy-time relationship of warfarin in rats with a good safe profile, we divided forty rats into four groups (n = 10), and administered them with oral warfarin at the doses of 0.2, 0.3, 0.4, and 0.5 mg/kg/day for 7 days. The 0.9 mL of whole blood were collected from rat tail vein at 4 h post dosing, and immediately mixed with 0.1 mL 3.2% sodium citrate. The plasma was separated from whole blood after centrifugation at 1000 g for 15 min at 4 °C. Prothrombin time (PT) and INR of each plasma were measured by the coagulation analyzer (MC-2000, Germany) within 2 h after blood draw. To monitor the dynamic change of INR in rats, we measured PT and INR each day, and tried to keep the INR between 2.0 and 3.0 daily by administration of warfarin at 0.4 mg/kg/day, the dose used throughout the study unless otherwise stated.

### Pharmacodynamic interaction experiments between warfarin and GS

Rats were divided into seven groups (n = 10 per group). Group 1 received warfarin only, group 2–4 received warfarin for 1 week followed by 3-week warfarin plus GS (30, 100, 300 mg/kg/day dosed at 4 h after warfarin administration). Group 5–7 received GS at 30, 100, and 300 mg/kg/day to serve as the monotherapy control in order to watch GS effect on the PT and INR. Blood was collected from each rat every other day for the PT and INR measurements.

Interaction between warfarin and GS on K-carrageenan-induced thrombosis model was also evaluated in five groups of the rats. Group 1 was the control saline, group 2 received warfarin only throughout the experiment, and groups 3–5 received warfarin for 1 week followed by 3 weeks of warfarin plus GS at 30, 100, and 300 mg/kg/day. After the regimens, K-carrageenan (0.5 mg/kg) in saline was injected to rat tails at a site 13 cm from the tail tip. After 24 h of K-carrageenan injection, the length of infracted regions in rat tails was measured and compared between groups.

### Pharmacokinetic interaction between warfarin and GS measured by UPLC/MS/MS

To determine whether drug-drug interaction exists between warfarin and GS on pharmacokinetics, we developed and validated the UPLC-MS/MS method similar to that we described previously^31^. The UPLC-MS/MS analysis was conducted in the positive ion mode on a Waters H-class liquid chromatograph interfaced with XEVO-TQD Mass Spectrometer (Waters) equipped with heated capillary interface, electrospray ionization (ESI) source, and a tandem quadrupole mass detector. Separation of each component was performed on an ACQUITY UPLC BEH C18 column (1.7 μm, 50 mm × 2.1 mm, id, Waters, USA). The mobile phase was consisted of methanol and water containing 0.1% formic acid (90:10, v/v) at a flow rate of 0.2 mL/min. Quantitative analysis was performed using MRM of the transitions of m/z 309 → 162.9 for warfarin, 325 → 179.2 for 7-OH-warfarin, and 237 → 194.2 for carbamazepine (the internal standard), respectively, with a total running time of 3 min for each sample. The injection volume was 3 μL.

The rats were randomly divided into the control (saline) and GS (300 mg/kg/day for 18 days) groups (n = 10 per group). All rats, with or without 18-day GS pretreatment, received a single oral dose of warfarin (1 mg/kg), and their blood was collected via tail vein at 0.5, 1, 2, 3, 4, 6, 12, 24, and 48 h after the single warfarin administration in order to determine the intervention of GS on blood warfarin levels. All acquired plasmas were frozen at −80 °C until analysis by the UPLC/MS/MS. The pharmacokinetic parameters such as AUC, CL, C_max_, and T_max_ were analyzed as we described previously by the software of DAS 3.0 (Bio Guider Co., Shanghai, China)^32^.

### Investigate effect of GS on warfarin metabolism in rats

Four groups of rats received warfarin (0.4 mg/kg/day) for one week followed by three weeks of warfarin plus GS at 30, 100 and 300 mg/kg/day. The GS was given at 4 h after oral dosing of warfarin. Blood samples were collected at 4 h after administration of GS at the end of each week, and stored at −80 °C to determine blood concentrations of warfarin and its metabolist 7-OH-warfarin by using the UPLC/MS/MS method.

### Investigate interaction between GS and warfarin on coagulation factor activity

Rats received warfarin (0.4 mg/kg/day) alone for 7 days before they were given warfarin plus GS (30, 100, 300 mg/kg/day at 4 h after warfarin) for 3 weeks. Blood samples were collected every other day and stored at −80 °C. At the designed times, plasma was prepared for measuring vitamin k-dependent coagulation factors II, VII and protein Z according to the instructions of the ELISA kit (TSZ, USA) for measuring the coagulation factors at OD450 nm using the Infinite M200 Pro microplate reader (Tecan, Switzerland). We first measured activity of those coagulation factors affected by the 1-week warfarin plus 3-week warfarin-GS regimen to determine the dose-dependent effect of GS (30, 100, 300 mg/kg/day) at the end of 3-week regimen. We then measured the time-dependent effect of GS on warfarin-induced inhibition of the coagulation factors by using the fixed dose of GS (300 mg/kg/day), and the dynamic change in activity of the coagulation factors was measured every week during the experiment.

### Investigate interaction between GS and warfarin on activity of rat liver cytochrome P450

Following the 1-week warfarin plus 3-week warfarin-GS regimen, we sacrificed the rats and excised the livers to make liver homogenization (3 mg tissue) with 1 mL TRIZOL reagent (Takara, Japan) and a tissue grinding apparatus. The total RNA was extracted and purified following the instruction by the manufacturer for the preparation of cDNA sample. The liver cytochrome P450 isomer cDNAs were synthesized by reverse transcription reagent (TaKaRa, China). The primers of P450 isomers CYP1A2, CYP2C9 and CYP3A4 were designed as 5′-CGCCCAGAGCGGTTTCTTA-3′ and 5′-TCCCAAGCCGAAGAGCATC-3′, 5′-GGACAGAGACGACAAGCACA-3′ and 5′-CATCTGTGTAGGGCATGTGG-3′, 5′-AAGTCGCCTCGAAGATACACA-3′ and 5′-AAGGAGAGAACACTGCTCGTG-3′, respectively, and applied to analyze the mRNA expression of the isomers during the *in vivo* warfarin and GS interaction by using the real-time PCR detection system (BioRad, USA).

### Statistical analysis

Data were expressed as the mean ± SD. Statistical analysis was performed with Student’s *t*-test for paired observation (two tailed). A probability (*P*) value of 0.05 or less was considered significant, and *P* of 0.01 or less was highly significant.

## Electronic supplementary material

Supplementary Information
