# Supplementary material for: Global deregulation of ginseng products may be a safety hazard to warfarin takers: solid evidence of ginseng-warfarin interaction
Source: Sci Rep. 2017 Jul 19;7:5813. doi: 10.1038/s41598-017-05825-9 (PMC5517508; doi:10.1038/s41598-017-05825-9)

Global deregulation of ginseng products may be a safety hazard to warfarin takers: solid evidence of ginseng-warfarin interaction

Haiyan Dong[#], Ji Ma[#], Tao Li, Yingying Xiao, Ning Zheng, Jian Liu, Yu Gao, Jingwei Shao, Lee Jia*

Cancer Metastasis Alert and Prevention Center, and Pharmaceutical Photocatalysis of State Key Laboratory of Photocatalysis on Energy and Environment, College of Chemistry; Fujian Provincial Key Laboratory of Cancer Metastasis Chemoprevention and Chemotherapy, Fuzhou University, Fuzhou 350116, China.

***Corresponding author:** Lee Jia (pharmlink@gmail.com or cmapcjia1234@163.com), Sunlight Building, 6FL; Science Park, Xueyuan Road, University Town; Cancer Metastasis Alert and Prevention Center, Fuzhou University, Fuzhou, Fujian 350116, China. Phone: +86-0591-2286-7183.

#, equally contributed to the work.

Suppl. Fig. S1 LC/MS/MS spectra of warfarin and 7-hydroxywarfarin

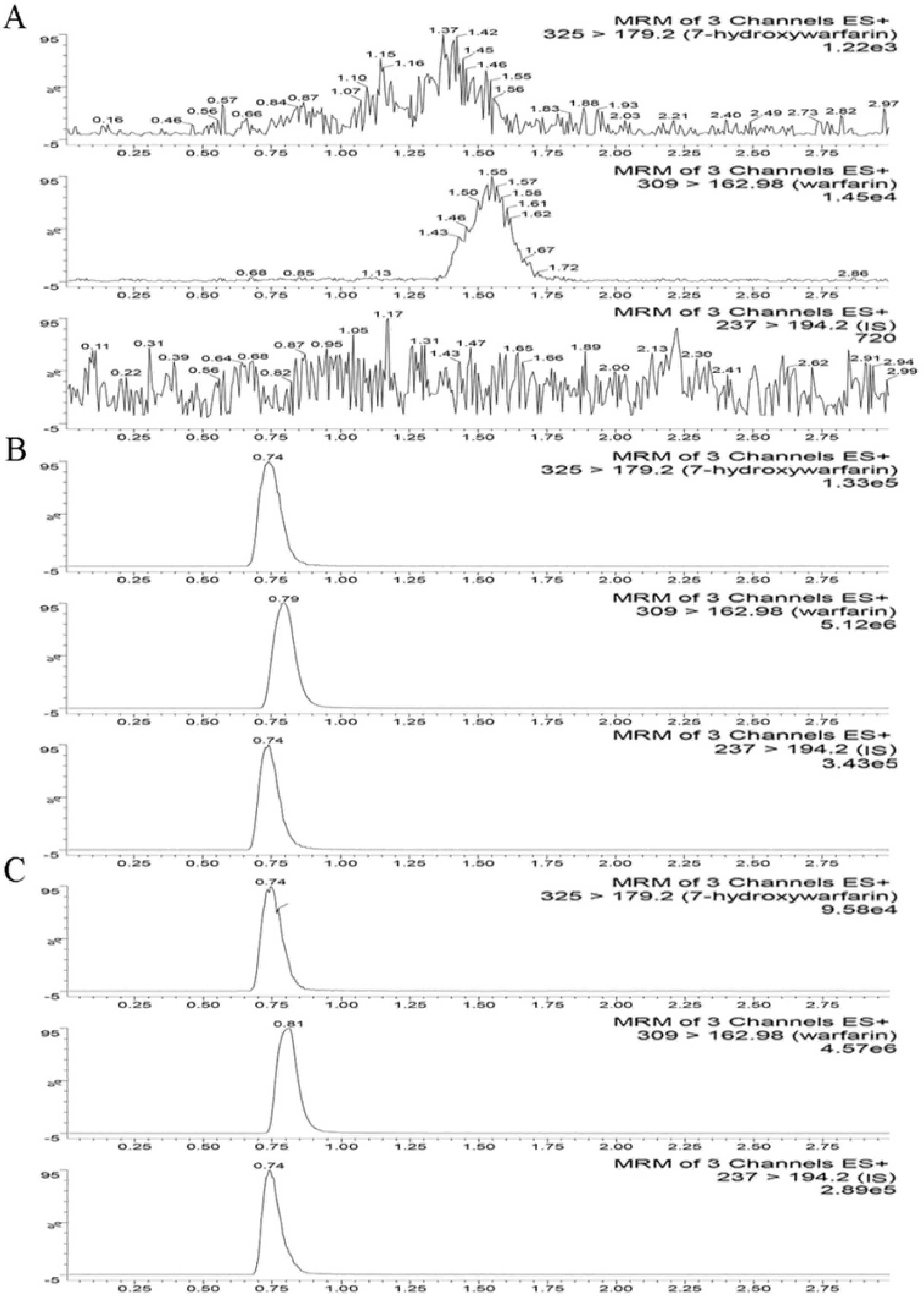

Supplement: Supplementary file 1 — Supplementary Information [file 41598_2017_5825_MOESM1_ESM.pdf]
